# Methodologies underpinning polygenic risk scores estimation: a comprehensive overview

**DOI:** 10.1007/s00439-024-02710-0

**Published:** 2024-10-19

**Authors:** Carene Anne Alene Ndong Sima, Kathryn Step, Yolandi Swart, Haiko Schurz, Caitlin Uren, Marlo Möller

**Affiliations:** 1 Division of Molecular Biology and Human Genetics, Faculty of Medicine and Health Sciences, South African Medical Research Council Centre for Tuberculosis Research, Stellenbosch University, Cape Town, South Africa; 2 Centre for Bioinformatics and Computational Biology, Stellenbosch University, Cape Town, South Africa

## Abstract

Polygenic risk scores (PRS) have emerged as a promising tool for predicting disease risk and treatment outcomes using genomic data. Thousands of genome-wide association studies (GWAS), primarily involving populations of European ancestry, have supported the development of PRS models. However, these models have not been adequately evaluated in non-European populations, raising concerns about their clinical validity and predictive power across diverse groups. Addressing this issue requires developing novel risk prediction frameworks that leverage genetic characteristics across diverse populations, considering host-microbiome interactions and a broad range of health measures. One of the key aspects in evaluating PRS is understanding the strengths and limitations of various methods for constructing them. In this review, we analyze strengths and limitations of different methods for constructing PRS, including traditional weighted approaches and new methods such as Bayesian and Frequentist penalized regression approaches. Finally, we summarize recent advances in PRS calculation methods development, and highlight key areas for future research, including development of models robust across diverse populations by underlining the complex interplay between genetic variants across diverse ancestral backgrounds in disease risk as well as treatment response prediction. PRS hold great promise for improving disease risk prediction and personalized medicine; therefore, their implementation must be guided by careful consideration of their limitations, biases, and ethical implications to ensure that they are used in a fair, equitable, and responsible manner.

## Introduction

Polygenic risk scores (PRS) are often thought of as new since they are derived from genome-wide association studies (GWAS); which is a relatively new concept. However, they have a deep and rich history stretching back over half a century. In 1967, Irving Gottesman and James Shields published “*A Polygenic Theory of Schizophrenia*”, providing early insights into the polygenic inheritance of complex traits, notably schizophrenia (Gottesman and Shields, 1967). Their seminal work revealed that while schizophrenia demonstrates high heritability (up to 80%), its transmission does not conform to classical Mendelian segregation, suggesting a polygenic susceptibility. Moreover, the study highlighted the influence of trait, population, and environmental factors on heritability. This work was remarkably ahead of its time with respect to the current understanding of human genetics. In 2001, Meuwissen and colleagues used a dense marker map coupled with a Bayesian approach to accurately predict breeding values in simulated genome data of animals with no phenotype and no progeny (Meuwissen et al., 2001).

The results highlighted that the best predictive model for estimated breeding values showed a strong correlation with true breeding values (r ± standard error = 0.848 ± 0.012), indicating high selection accuracy. For comparison, pedigree-based selection yields a lower accuracy of around 0.4. This was the first study that demonstrated the use of the additive effect of genetic markers to predict phenotypic outcome. This study ushered the widespread use of the same polygenic risk approaches that are described today in humans, animals, and plant breeding. Another seminal paper from International Schizophrenia Consortium et al. (2009) demonstrated empirically the findings of the previous two papers (International Schizophrenia Consortium et al., 2009). Genetic scores were calculated using all SNPs at p-value < 0.5 from 3322 European individuals with schizophrenia and 3587 controls. These scores were higher in cases and showed a strong correlation with phenotypic outcomes, explaining approximately 3% of variance. The results suggested, for the first time in a complex human disease and remarkably in the context of a GWAS with no significant associations, that one could potentially predict case/control status in a new sample simply from the additive sum of single nucleotide polymorphism (SNP) weights. This discovery was a significant breakthrough with respect to the potential of polygenic variation and its ability to not only predict disease outcomes but also detect shared genetic aetiology between traits. The findings also presaged both the transferability of the polygenic scores across different global populations and the concept of “missing heritability”, which is when the phenotypic variance (i.e., SNP-based heritability) explained by a PRS model is less than the known/suspected heritability of a disease (Choi et al., 2020). However, it has been shown that the issue of “missing heritability” should be alleviated as GWAS sample sizes increase (Dudbridge, 2013; Machiela et al., 2011). This suggests that as GWAS sample sizes increase, so will PRS predictive power until it reaches the limit determined by trait heritability (Choi et al., 2020).

Despite the significant strides made in genetic risk prediction and their potential benefits, several challenges also hinder their implementation in clinical settings. In this review, we analyze the strengths and limitations of different methods for constructing PRS, including traditional weighted approaches and new methods such as Bayesian and Frequentist penalized regression approaches. Additionally, we summarize recent advances in the development and application of PRS, focusing on their potential research and clinical applications. Finally, we highlight key areas for future research, including the need to develop PRS models that are robust across diverse populations by underlining the complex interplay between genetic variants across diverse ancestral backgrounds in disease risk as well as treatment response prediction. PRS hold great promise for improving not only disease risk prediction but also treatment outcomes and personalized medicine; therefore, their implementation must be guided by careful consideration of their limitations, biases, and ethical implications to ensure that they are used in a fair, equitable, and responsible manner.

## From genome-wide association studies (GWAS) to polygenic risk scores (PRS)

### Genome-wide PRS methods

GWAS have been successful at identifying common SNPs associated with complex phenotypes such as, but not limited to, cardiovascular diseases, cancers, neurodegenerative diseases as well as infectious diseases like tuberculosis (Abdellaoui et al., 2023; Andrews et al., 2023; Bashinskaya et al., 2015; Blauwendraat et al., 2020; Hameed et al., 2024; Liang et al., 2020; Ndong Sima et al., 2023; Nott and Holtman, 2023; Walsh et al., 2023). Although significant associations have been found, each SNP on its own has a small effect on a disease outcome. This could be explained by the common-disease-common-variant (CDCV) hypothesis which states that if a disease is common in a population (prevalence > 1–5%), then its genetic contributors will also be common in that population with small individual effect size. Therefore, the trait/disease outcome is believed to be due to the cumulative effect of multiple variants (International Schizophrenia Consortium et al., 2009; Lango Allen et al., 2010; Pereira et al., 2021).

PRS construction and analysis can be divided into three main steps: (1) quality control of data, (2) calculation of the scores, and (3) PRS performance assessment. The first two steps typically requires two independent input datasets: (i) a discovery dataset (i.e., SNPs summary statistics), from which SNPs and their subsequent effect sizes are obtained and (ii) a target dataset (i.e., cohort understudy), from which genotype dosage of each SNP included in the calculation are obtained. For the third step, the performance of the predictive models is assessed either using cross-validation methods (generally if the target sample size is too small to be split) or an independent dataset (i.e., testing set). Each step is outlined as follows:

#### Quality control of discovery and target data

The basis of PRS is a SNPs summary statistics which can be obtained either from GWAS or candidate genes association studies. Therefore, the initial step is quality control of input data which is performed similarly to a standard GWAS quality control (as described by (Marees et al., 2018)). In addition, to avoid systematic errors, users must confirm that all datasets are aligned to the same genome build. This is to ensure that all datasets have not only SNPs that have the same genomic positions and SNP identifiers (i.e., rsID), but also share the same SNPs. Furthermore, it is important to remove ambiguous SNPs (A/T and/or C/G). When SNPs have complementary alleles, it becomes challenging to distinguish which of the strands is being measured and therefore, which is the effect allele (Choi et al., 2020). This could therefore be a major source of systematic error. Sometimes, it is possible to solve this ambiguity using information on allele frequency, but this can be daunting if the allele frequencies of those SNPs are close to 0.5 (Chen et al., 2018). Therefore, general practice is to remove these ambiguous SNPs. It should also be ensured that discovery and target samples are independent from one another, to avoid the issue of overfitting that is inherent in machine learning (Choi et al., 2020).

#### Calculation of traditional polygenic risk scores: which variants to include and how to account for linkage disequilibrium?

**Fig. 1:**
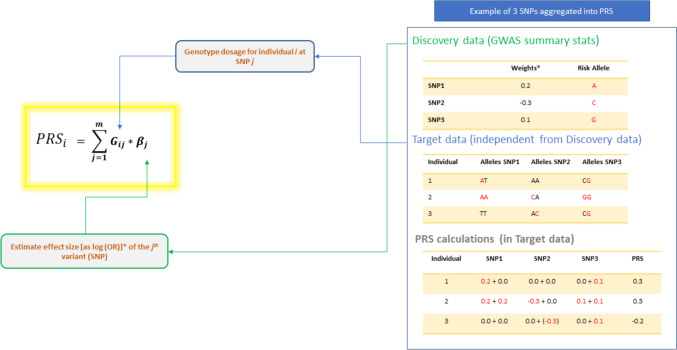
Graphical illustration of polygenic risk score calculation where variants weights are obtained either directly from a GWAS summary statistics or corrected by accounting for LD between them. Regardless of how the SNPs weights are estimated, for each selected SNP, the weight is multiplied by the number of effect (risk) alleles (in red) and summed over all variants to get a polygenic score

PRS are calculated as the sum of an individual’s risk alleles, weighted by their effect sizes (Chatterjee et al., 2013; Choi et al., 2020; Lango Allen et al., 2010; Pharoah et al., 2008). This can generally be summarized as follows and illustrated as in Fig. 1 below:

1 $$\:{PRS}_{i}=\:\sum\limits_{j=1}^{m}{G}_{ij}{*\beta\:}_{j}$$

Where the PRS for individual (*i*) is the sum of the genotype dosage (*G*) for each SNP (*j* to *m*) multiplied by the effect *βj* for that allele.

Ideally, all the SNPs should be summed across all loci. However, there are two factors to consider: (1) many GWAS are underpowered, which means that there could be more true associations than those discovered (i.e., those reaching genome-wide significance); (2) the inherent issue of linkage disequilibrium (LD), which creates a correlation structure among nearby variants and can, inflate the PRS and result in poor generalizability across populations (Dudbridge, 2013; Duncan et al., 2019; Meuwissen et al., 2001). In the hope to curb the inflation of PRS only independent markers should be used (Meuwissen et al., 2001; Vilhjálmsson et al., 2015).

#### PRS model performance assessment

The assessment of the PRS performance is crucial for their application in clinical settings. The evaluation typically involves measure of predictive performance such as squared correlation (R^2^) or Nagelkerke’s pseudo-R^2^ and the area under the receiver operating characteristic curve (AUC) (Choi et al., 2020). The former two quantify how much of the variation in a trait among individuals can be explained by their genetic makeup (referred to as proportion of phenotypic variance), whereas the latter is a composite of sensitivity and specificity (with maximum value of 1.0), and can be used to determine how well a model can distinguish between individuals at high risk and those at low risk for a particular disease. Additionally and of equal importance is calibration of the scores as it ensures that the predicted risks align with observed outcomes in the population. The necessity of calibrating PRS has been highlighted before clinical implementation, as miscalibrated scores could lead to inappropriate risk assessments (Vilhjálmsson et al., 2015; Wei et al., 2022). For instance, a study by Wei and colleagues showed a systematic bias between estimated risks values and observed risks for the prostate cancer, breast cancer, and colorectal cancer in three incident cohorts from the UK Biobank (β (95% CI) was 0.67 (0.58–0.76), 0.74 (0.65–0.84) and 0.82 (0.75–0.89) respectively) which was significantly lower than the expected value of 1.00 under perfect calibration (Wei et al., 2022).

### Traditional approach to PRS calculation: clumping and thresholding |

The traditional approach to PRS calculation identifies near independent SNPs using a method called clumping and thresholding (C + T). Briefly, clumping involves sorting SNPs by importance (from the most significantly associated markers to the least) where the most important SNP is tagged as the “index” of the predefined non-overlapping window and the other correlated SNPs are removed. As opposed to pruning, this procedure ensures that the index SNP is never removed, keeping at least one representative SNP for each region of the genome (Privé et al., 2019). The analysis proceeds with the next most significant SNP that has not been removed yet. In that fashion, the SNPs with the strongest signals (lowest p-value) are maintained, allowing for the construction of a more predictive genetic score. Therefore, user-driven choices of variants correlation values (as cut-off values) will consequently play a role in the prediction accuracy of the scores.

After clumping, genetic variants are approximately independent. However, the question of how significant the association needs to be for inclusion in the PRS calculation remains unanswered. One proposed solution is p-value thresholding which calculates PRS at various p-value thresholds. This method implies that the optimising parameters to compute the best predictive PRS model are a priori unknown (Choi et al., 2020). Therefore, PRS are calculated over a range of p-values and the most predictive one is chosen using a measure of predictive performance such as Nagelkerke’s pseudo-R^2^ or R^2^ (Choi et al., 2020). The C + T approach is implemented in various software packages such as PLINK, PLINK2, PRSice, and PRSice-2 (Chang et al., 2015; Choi and O’Reilly, 2019; Euesden et al., 2015; Purcell et al., 2007). While PRSice must be run several times for different p-value thresholds, PRSice2 has a more automated system for the thresholding approach. The C + T approach is an attractive method for PRS calculation as it is user friendly, not computationally extensive and with results that are easy to interpret. However, the C + T method has several flaws in that by applying single optimal cut-off values, the approach assumes similar LD patterns throughout the genome. This creates a misrepresentation of SNPs, especially within long-range LD regions of the genome (Privé et al., 2021). Additionally, this method does not take into consideration the intrinsic genetic differences that can exist between the discovery and target datasets even if both are subsets of the same population. This is especially true for African populations or admixed individuals who exhibit high levels of genetic heterogeneity across and within population. This phenomenon often results in missed association signals and therefore skewed polygenic scores.

### Non-traditional approaches to PRS calculation

In recent years, many approaches to PRS calculations have investigated the inclusion of all SNPs while accounting for LD between them, thereby calculating LD-corrected variants weight (see Table 1). Unlike the C + T approach which uses the marginal effect sizes of variants (meaning the effect size of each SNP without considering the correlation between them), these methods intend to model the joint effect of all SNPs. Two approaches have mainly been explored: (1) Bayesian penalized regression, and (2) Frequentist penalized regression. Although the two approaches differ fundamentally in their underlining assumptions (e.g., prior distributions, L1 regularization, etc.) and their interpretation of uncertainty, they share common goals and methodologies in improving model performance through regularization techniques. These approaches are particularly useful for variable selection and addressing issues like overfitting, which can arise when the number of predictors exceeds the number of observations.

#### Bayesian penalized regression approach

The Bayesian framework leverages prior distributions and advanced modelling techniques to enhance prediction accuracy while maintaining interpretability (Ge et al., 2019; Hu et al., 2017; Lloyd‑Jones et al., 2019; Vilhjálmsson et al., 2015); and the selection of those priors are made in a way that best capture the genetic architecture of the trait of interest.

The older method, LDpred utilizes a point-normal prior for modelling the SNPs effect sizes (Vilhjálmsson et al., 2015). This assumes that there is a proportion (*p*) of causal variants on a given trait of interest and that their joint effect sizes are normally distributed with mean zero and variance proportional to the heritability (h_2_) of the trait. Importantly, ***p*** and h_2_ are estimated independently. For ***p***, a grid of values is explored similarly to the p-value thresholding used in C + T; whereas h_2_ or SNP-based h_2_ is estimated using LD score regression. Then, a Bayesian Gibbs sampler is used to estimate the joint effect sizes of SNPs in the GWAS summary statistics (i.e., the discovery dataset) by accounting and modelling a matrix of the LD pattern from an external reference data. The LDpred model has been shown to outperform the traditional C + T approach (Khera et al., 2018; Vilhjálmsson et al., 2015). One example is a study by Vilhjálmsson and colleagues who compared the performance of LDpred to C + T across a range of diseases and trait (schizophrenia (SCZ), multiple sclerosis (MS), breast cancer (BC), type 2 diabetes (T2D), coronary artery disease (CAD), and height). The results showed that LDpred provided significantly better predictions than other approaches with the relative increase in Nagelkerke R^2^ ranging from 11% for T2D to 25% for SCZ, and a 30% increase in prediction R^2^ for height; the p-values were 6.3 × 10^−47^ for SCZ, 2.0 × 10^−14^ for MS, 0.020 for BC, 0.004 for T2D, 0.017 for CAD, and 1.5 × 10^−10^ for height (Vilhjálmsson et al. 2015). Similar trends were consistently replicated in different benchmarking studies (Lloyd-Jones et al. 2019; Khera et al. 2018).

Similarly to LDpred, the SBayesR software programme (Lloyd‑Jones et al., 2019) also uses point-normal distribution. However, it combines a likelihood function that connects the joint effect sizes with the GWAS summary statistics coupled with a finite mixture of normal distribution priors underlying the variant effects. This basically means that the SNP effect sizes are modelled as a mixture of normal distributions with mean zero and different variances. The modelling is typically done using four normal distributions all with mean zero and distinct variances. The first one is variance zero, which captures all the SNPs with a zero effect and from there, increasing values of effect sizes are allowed to exist in the model. Using summary statistics for variants from the largest GWAS meta-analysis on height and BMI (*n* = 700,000), Lloyd-Jones and colleagues demonstrated that on average across traits and two independent datasets that SBayesR improves prediction R^2^ by 5.2% compared to LDpred and by 26.5% compared to C + T (Lloyd-Jones et al. 2019). Although SBayesR performs better than the LDpred model and the C + T approach, it has the same pitfalls as the former model. In that sense, they both remain unstable in long-range LD regions of the genome and therefore severely underperform for autoimmune diseases such as Type 1 diabetes (T1D) and rheumatoid arthritis (RA) (Lloyd-Jones et al. 2019; Privé et al. 2021; Márquez-Luna et al. 2021). Additionally, both LDpred and SBayesR do not model functional enrichment of causal variants effect sizes which when accounted for, has been shown to improve polygenic prediction accuracy (Márquez‑Luna et al., 2021).

In that effect, Márquez-Luna and colleagues proposed LDpred-funct, an extension of LDpred, to estimate posterior distribution of causal effect sizes by accounting for LD between SNPs and leveraging trait-specific functional priors, which are designed based on the biological relevance of genetic variants (Márquez‑Luna et al., 2021). This approach recognizes that variants located in functional regions (e.g., coding, regularoty, or conserved regions) are more likely to contribute to complex phenotypes. The results showed a 4.6% relative improvement in average prediction accuracy (average R^2^ = 0.144 for 21 highly heritable traits in the UK Biobank; highest R^2^ = 0.413 for height) compared to SBayesR (Márquez‑Luna et al., 2021). Thus, highlighting the relative gain in predictive power when incorporating functional priors, consistent with the functional architecture of the complex trait understudy.

Another software tool is PRS-CS that employs a continuous shrinkage (CS) prior on SNP effect sizes which allows for adaptive shrinkage, where the amount of shrinkage can vary across coefficients based on the strength of association signals from GWAS (Ge et al., 2019). This adaptability makes PRS-CS robust to varying genetic architectures across different traits and populations. Additionally, the method utilizes summary statistics from existing GWAS rather than requiring individual-level genotype data. This makes it feasible to apply it in large-scale studies where individual-level data may not be available. Using data from the Partners Biobank, Ge and colleagues observed an average improvement of 18.17% and 11.41% across all trait when using PRS-CS and PRS-CA-auto (respectively) compared to LDpred. This improvement was around 3-fold increase relative to C + T (Ge et al., 2019).

A newer method, LDpred2 (Privé et al., 2021) is a recent update to LDpred with two new modes added. The first one, LDpred2-auto, estimates ***p*** and h_2_ directly from the model, instead of testing several values and using LD score regression (Privé et al., 2021). The other mode, LDpred2-sparse (option from the -grid model), allows for effect sizes to be exactly zero, similarly to the first mixture component of SBayesR. Additionally, LDpred2 addresses instability issues that were present in earlier methods by providing a more stable workflow and by modelling long-range LD region such as that found near the HLA region of chromosome 6 (Evseeva et al., 2010; Privé et al., 2021). Some of the earlier methods rely on removing these regions to account for the problem (Lloyd‑Jones et al., 2019; Márquez-Luna et al., 2021; Vilhjálmsson et al., 2015), which has been shown to reduce prediction accuracy since these regions harbour many immunity-related genes (Evseeva et al., 2010; Privé et al., 2021). LDpred2 has been benchmarked against other polygenic score methods, demonstrating a slight predictive performance improvement (Privé et al., 2021). For instance, it achieved a mean AUC of 65.1% across multiple traits from the UK Biobank against 63.8% for lassossum, 62.9% for PRS-CS, and 61.5% for SBayesR.

#### Frequentist penalized regression approach

In Frequentist penalized regression framework, the goal is to minimize a penalized loss function that combines the model’s error with a penalty that discourage large coefficients. However, in this framework unlike in the Bayesian’s, SNP weights are shrunk based on a penalty term but there is no notion of prior beliefs about parameters. This method applies a penalty directly to the regression coefficients, which helps to mitigate issues such as collinearity and overfitting, thereby enhancing model interpretation and prediction accuracy. For example, ridge regression is known for its ability to handle multicollinearity by imposing a penalty on the size of the coefficients, while Least Absolute Shrinkage and Selection Operator (LASSO) can shrink some coefficients to zero, effectively performing variable selection (Cule and De Iorio, 2013). The elastic net combines both LASSO and ridge penalties, providing a balanced approach that can be particularly useful when predictors are highly correlated (Kohannim et al., 2012; Tessier et al., 2015).

The software programme Lassosum was the first to use LASSO regression to shrink marker effects (Mak et al., 2017). This approach makes LASSO regression robust against the issue of nonconvergence which is inherent in programmes that use a Bayesian method (Mak et al., 2017). In addition, Lassosum has been reported to not only surpass the performance of both LDpred and the C + T method, but also demonstrate superior computational speed. The latter is a desired attribute, especially as GWAS sample size increases. A recent update to Lassosum is Lassosum2 (Privé, Arbel, Aschard, et al., 2021), which has been re-implemented in the R package *bigsnpr* and uses the same input data as LDpred2 with no additional coding nor computational time. Lassosum2 can therefore be used while already running LDpred2 with no loss on the predictive performance. As PRS is gaining momentum due to the rise of GWAS, algorithm developments are crucial to ensure that genetic prediction achieves its full potential.

### Pathway-based PRS method

Evidently, a great amount of effort is being put into the development and optimisation of PRS calculation tools. Ideally, these approaches could help stratify individuals based on their relative risk of developing a particular disease. However, genome-wide PRS methods cannot always provide great insights into the heterogeneity of complex disease (Broekema et al., 2020; Choi et al., 2023; Visscher et al., 2021). In fact, genome-wide PRS are dominated by variants that affect multiple disease sub-types, which means that unless deep phenotyping of a disease/trait is generated prior to association testing, the genome-wide aggregation of effects reduces variants’ specificity (Choi et al., 2023). Therefore, these methods are limited-by-design in their inability to stratify individuals into disease sub-types. To circumvent the issue, Choi and colleagues propose PRSet, the first pathway-based PRS approach and software (Choi et al., 2023). The tool was designed to calculate PRS by incorporating variants with significant genetic signal at pathway-level and variants in regions which have higher heritability. This therefore provides a genetic risk prediction method that accounts for an individual’s more complete genetic profile. PRSet would therefore be able to further stratify individuals into groups of more homogenous disease sub-types and thus offering a proof-a-principle of their potential utility to provide more powerful paths to precision medicine (Choi et al., 2023).

To test their theory, Choi and colleagues investigated the performance of genome-wide PRS versus pathway-PRS to stratify individuals into disease sub-types (Choi et al., 2023). The results showed that while the discriminatory power for the classification subtypes was overall low, PRSet consistently performed better than Lassosum and PRSice. The median R^2^ estimate using PRSet was 9.27 × 10^−3^ for discriminating Crohn’s disease vs. Ulcerative colitis, and 0.032 for Bipolar disorder I vs. Bipolar disorder II. Next, the performance of the standard application of PRS in predicting phenotypic outcome was evaluated. Using four well-established diseases from samples of the UK Biobank (Type 2 diabetes (T2D), coronary artery disease, low-density lipoproteins (LDL), and obesity) the results showed that the relative improvement in performance for PRSet vs. Lassosum and PRSice was reduced relative to the stratification analyses, and in the case of obesity, Lassosum performed slightly better than PRSet (R^2^ = 0.042 vs. 0.039, respectively). For the four traits, the phenotypic variance explained by PRSice was the lowest. Although the study provides evidence that PRSet is a promising method for improving PRS accuracy, the authors do not presently recommend PRSet over well-established genome-wide PRS methods. This is mainly due to major limitations of the model pertaining to the lack of current knowledge on (1) disease sub-types specific pathways and (2) SNP-pathway association.

**Table 1:** Summary of some past and current PRS tools

PRS Tool	Methodology^1^	Data requirements	Implementation	Source	Website/GitHub repository
PLINK (2007)	C + T (manual thresholding)	GWAS summary statistics	PLINK framework	(Purcell et al., 2007)	https://www.cog-genomics.org/plink
PLINK2 (2015)				(Chang et al., 2015)	https://www.cog-genomics.org/plink2
PRSice (2015)	C + T	GWAS summary statistics	R and C++	(Euesden et al., 2015)	https://github.com/choishingwan/PRSice
LDpred (2015)	Bayesian regression	GWAS summary statistics	Python based	(Vilhjálmsson et al., 2015)	https://github.com/bvilhjal/ldpred
Lassosum (2017)	Lasso penalized regression	GWAS summary statistics or individual-level data	R package: *lassosum*	(Mak et al., 2017)	https://github.com/tshmak/lassosum
PRSice-2 (2019)	C + T	GWAS summary statistics	R and C++	(Choi and O’Reilly, 2019)	https://choishingwan.github.io/PRS-Tutorial/prsice/
PRS-CS (2019)	Bayesian regression with continuous shinkage priors	GWAS summary statistics and LD reference panel	Python based	(Ge et al., 2019)	https://github.com/getian107/PRScs
SBayesR (2019)	Bayesian multiple regression	GWAS summary statistics	GCTB software	(Lloyd‑Jones et al., 2019)	http://cnsgenomics.com/software/gctb
LDpred-funct (2019)	Bayesian regression	GWAS summary statistics	Python package	(Márquez‑Luna et al., 2021)	https://github.com/carlaml/LDpred-funct
LDpred2 (2020)	Bayesian regression	GWAS summary statistics and LD reference panel	R package: *bigsnpR*	(Privé, Arbel, and Vilhjálmsson, 2021)	https://privefl.github.io/bigsnpr/articles/LDpred2.html
Lassosum2 (2021)	Lasso penalized regression	GWAS summary statistics or individual-level data	R package: *bigsnpR*	(Privé, Arbel, Aschard, et al., 2021)	https://privefl.github.io/bigsnpr/reference/snp_lassosum2.html
PRS-CSx (2022)	Bayesian regression with shared continuous shrinkage priors	Multiple GWAS summary statistics	Python based	(Ruan et al., 2022)	https://github.com/getian107/PRScsx
BridgePRS (2023)	Bayesian regression	GWAS summary statistics	Shell script	(Hoggart et al., 2023)	https://github.com/clivehoggart/BridgePRS
PRSet (2023)	Pathway-based	GWAS summary statistics	R and C++	(Choi et al., 2023)	Currently under active development but can be downloaded for free under the PRSice website
GAUDI (2024)	Fused lasso	GWAS summary statistics	R, Python, Shell script	(Sun et al., 2024)	https://github.com/quansun98/GAUDI

^1^C+T: (LD-) Clumping and Thresholding; lasso: Least Absolute Shrinkage and Selection Operator

### Improving prediction accuracy for underrepresented populations and admixed individuals

**Fig. 2:**
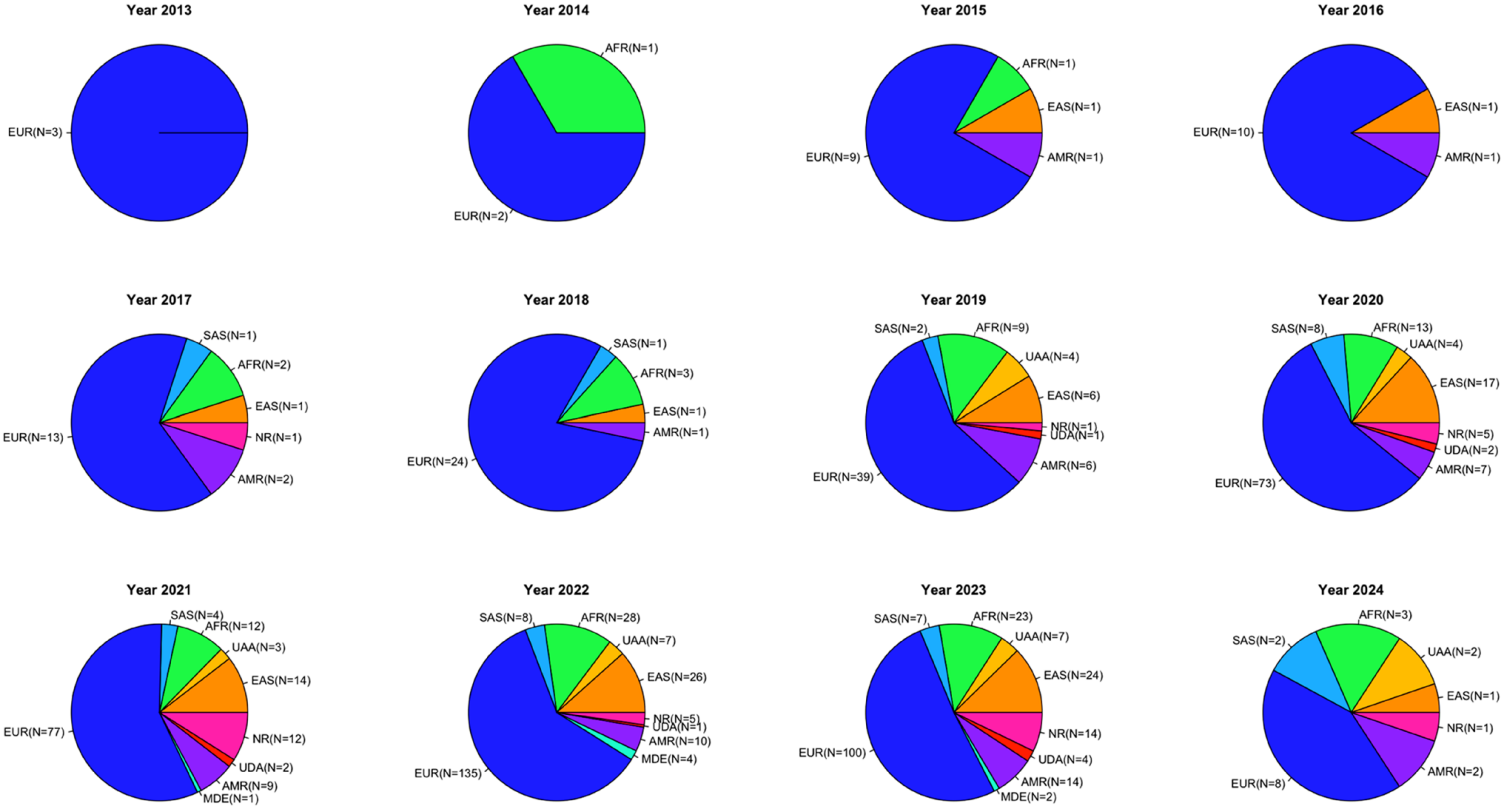
Results of all reported studies in the PGS Catalog depicting the prevalence of the use of diverse global populations in polygenetic risk score studies per year from 2013 to 2024. The studies are available on the PGS Catalog website https://www.pgscatalog.org/ accessed in April 2024. *AFR* African; *AMR* Admixed American; *EAS* East Asian; *EUR* European; *MDE* Middle Eastern; *NR* Not reported; *SAS* South Asian; *UAA* Unspecified Asian Ancestry; *UDA* Unspecified Diverse Ancestry

The studies presented above provide evidence for the development and optimisation of PRS methods to stratify individuals by risk of developing a given disease. However, they have mainly been assessed in populations of European ancestry; consistent with the dominance of Europeans in large-scale genomic studies. Figure 2 illustrates the prevalence of different ancestral groups in PRS for a better visualization of the disparities observed in genetic prediction studies. To reiterate, this represents a key obstacle to PRS portability as genetic insights from European-ancestry populations have been shown to have limited transferability to other global populations and vice-versa (Bitarello and Mathieson, 2020; Fatumo et al., 2023; Grinde, Qi, et al., 2019; International Schizophrenia Consortium et al., 2009; Marnetto et al., 2020; Márquez‑Luna et al., 2017; Martin et al., 2017, 2019). To support this claim, multiple studies have demonstrated that genetic prediction accuracy would likely decay with increasing genetic divergence between the GWAS discovery population and the PRS-targeted population. This implies that the genetic distance between the discovery and target datasets is inversely proportional to the predictive value of PRS model. Case in point, most PRS achieve better performance accuracy in target samples of European ancestry but transfer poorly to other population groups especially to individuals of African descent (who are severely underrepresented in genomic studies) (Grinde, Qi, et al., 2019; Martin et al., 2018). This lack of portability has been credited to genetic drift resulting from the bottleneck effect during the “Out-of-Africa” expansion, variants frequency, and LD pattern differences (which are known to be population specific) as well as the effect of gene-gene and gene-environment interactions (Duncan et al., 2019; Martin et al., 2017). The resultants of this genetic drift would lead to a discovery bias towards more common variants in populations that the GWAS was conducted in. Therefore, to ensure a genuinely representative and equitable PRS in clinical setting, it is imperative to prioritize the simultaneous development of both methods and models, along with the recruitment and inclusion of more diverse populations in genomic studies (Cavazos and Witte, 2021; Khera et al., 2018; Martin et al., 2017).

### Software development in polygenic prediction of underrepresented populations

Although a step towards alleviating the Euro-centric bias in PRS studies has been observed with the expansion of PRS utility to populations of Asian ancestry (mostly East Asians), African, and admixed individuals remain severely underrepresented (Duncan et al., 2019; Fatumo et al., 2023; Pereira et al., 2021). Therefore, the best approach to accurately predict disease risk in these populations remains unclear, reducing PRS transferability even further. Researchers have begun to explore various approaches for developing improved PRS tools for population groups who are historically underrepresented in genomic studies. These models include leveraging large-scale multi-ethnic datasets as well as integrating ancestry-specific genetic information. In recent years, several notable tools have emerged, each aiming to address specific challenges associated with the transferability and accuracy of PRS derived from GWAS.

Ruan and colleagues proposed PRS-CSx, an extension of PRS-CS, which aims to enhance cross-population polygenic prediction by jointly modelling GWAS summary statistics from multiple populations (Ruan et al., 2022). PRS-CSx incorporates a shared continuous shrinking prior to linking SNP effects across populations, facilitating more accurate estimation of effect sizes by leveraging information from summary statistics and taking advantage of the diversity in LD across discovery samples. This shared prior allows for correlated yet variable effect size estimates across populations, retaining the flexibility of the modelling framework (Ruan et al., 2022). Furthermore, PRS-CSx explicitly accounts for population-specific allele frequencies and LD patterns, and inherits the computational advantages of continuous shrinkage (CS) priors as well as efficient posterior inference algorithms (such as Gibbs sampling) from PRS-CS. When provided with GWAS summary statistics and ancestry-matched LD reference panels, PRS-CSx calculates a polygenic score for each discovery sample and integrates them by learning the optimal linear combination to generate the final PRS. This Bayesian multi-discovery method has been shown to dramatically increased prediction accuracy relative to other Bayesian single-discovery methods (Ruan et al. 2022; Hassanin et al. 2023; Zhang et al. 2023). For example, Ruan and colleagues evaluated the predictive performance of different PRS methods using UK Biobank and Biobank Japan (BBJ) as discovery sets (Ruan et al. 2022). The results showed that when predicting into a European cohort, PRS-CSx provided a consistent but marginal improvement over LDpred2 (trained on East Asian cohort GWAS; median relative increase in R^2^: 4.7%) and PRS-CS (trained on European cohort GWAS; median relative increase in R^2^: 5.2%). When using an East Asian cohort as target, PRS-CSx showed a substantial improved accuracy with increases of 52.3% and 32.9% over LDpred2 and PRS-CS (trained on UK Biobank GWAS), and 69.8% and 74.4% over LDpred2 and PRS-CS (trained on BBJ GWAS). Even in a case where neither discovery datasets matched the target (e.g., African cohort), PRS-CSx improved accuracy by 45.1% and 16.9% over LDpred2 and PRS-CS (Ruan et al., 2022). The study also highlight that multi-ancestry PRS were generalizable across different populations, effectively capturing genetic effects that ancestry-specific models might miss. Thus, showing the advantage of trans-ancestry modeling. It is important to note however that PRS-CSx has some pitfalls. While it can accommodate any number of GWAS summary statistics, it requires an ancestry-matched LD reference panel for each discovery sample, which can be challenging to construct. Additionally, PRS-CSx employs a fine-mapping approach to identify causal variants by utilising Bayesian modelling, reducing the number of candidate SNPs and improving our understanding of context-specific variants that contribute to a trait (Broekema et al., 2020). Although this is advantageous, fine-mapping approaches may be less effective when causal variants are either missing or lack sufficient statistical power for identification.

To address the limitations of PRS-CSx, a newer method called BridgePRS was developed (Hoggart et al., 2023). This novel Bayesian approach aims to enhance the accuracy of PRS in non-European populations by leveraging shared genetic effects across ancestries. BridgePRS combines information from a well-powered GWAS conducted in a discovery population (not matched to the target sample’s ancestry) with a second GWAS of limited power conducted in a dataset well-matched to the target dataset’s ancestry. This approach is similar to the linear combination of PRS which demonstrated improved PRS performance compared to single-population PRS approaches (Márquez‑Luna et al., 2017). In a three-stage iterative process, BridgePRS first trains and optimizes a PRS using data from a well-powered discovery population. To account for uncertainty in the location of causal variants, SNP effects are averaged across potential loci, assuming a zero-centred Gaussian prior distribution for SNP effect sizes. In the second stage, these SNP effect sizes serve as priors and are updated in a Bayesian framework using the GWAS data from the target population, such as individuals of African ancestry. Finally, ridge regressions are performed to obtain adjusted PRS estimates and weights, which are aggregated to generate the final PRS. Briefly, ridge regression works by adding a “penalty” term to the traditional linear regression model. This penalty encourages the model to keep the coefficients (i.e., the numbers that determine the importance of each feature or variable) small, which, in turn, makes the model less likely to fit the noise in the training data. This constraint helps the model generalise better to new data because it does not rely too heavily on the peculiarities of the training data.

The iterative process of prior-posterior updates allows BridgePRS to achieve fast and efficient analytical processing while demonstrating superior performance compared to other PRS methods in predicting disease risk in non-European population (Hoggart et al., 2023). In a study involving individuals of African, South Asian, and East Asian ancestries, BridgePRS exhibited slightly better predictive performance than PRS-CSx for 19 different traits in individuals of African ancestry with a relative boost in R^2^ of 60% (Hoggart et al., 2023). However, for individuals of Asian ancestry, PRS-CSx showed better accuracy than BridgePRS. Generally, the trend indicated that the latter outperformed the former when there was higher uncertainty in the mapping of causal variants. BridgePRS also outperformed single-ancestry PRS methods adapted for trans-ancestry prediction in the study. Hoggart and colleagues recommend both BridgePRS and PRS-CSx, as they have complementary strengths, and the optimal choice between the two methods depends on the specific trait and study characteristics (Hoggart et al., 2023). However, further research is needed to determine which method offers greater power in a given setting.

### Methods development in polygenic prediction for admixed individuals

Improving polygenic prediction for admixed individuals presents a critical challenge and an important area of research. These individuals are the result of interbreeding between previously geographically isolated populations and as such, exhibit very complex LD patterns characterized by ancestral-induced LD as well admixture-induced LD (Duan et al., 2018; Swart et al., 2021). Therefore, the general PRS calculation method that utilizes only a single training population would not be adequate to predict polygenic risk in admixed individuals as the appropriate discovery cohort is unknown. To address this, a linear combination of PRS based on two training datasets was proposed aiming to improve prediction accuracy of the model in admixed populations with some level of European ancestry proportions (Márquez‑Luna et al., 2017). Márquez-Luna and colleagues predicted type 2 diabetes (T2D) in a Latino and South Asian cohorts using a European (*N* = 40,000), Latino (*N* = 8000), and South Asian (*N* = 16,000) data, achieving over a 70% relative improvement in prediction accuracy compared to methods using a single discovery dataset (Márquez‑Luna et al., 2017). Similar observations were also made in an African data for height prediction which showed a 30% improvement. The approach takes advantage of the accuracy that can be achieved from large, well-powered European datasets and datasets showing similar LD patterns as the target population (Chatterjee et al., 2013; Dudbridge, 2013).

In agreement with the linear combination approach, another model combined multiple ancestry-specific partial polygenic scores that leverages ancestry-specific effect sizes to mitigate the issue of PRS model transferability to admixed individuals (of European and African ancestry) while adjusting for local ancestry (Marnetto et al., 2020). However, although that approach only showed modest improvement in predictive power when compared to the method of Márquez-Luna et al. (2017), the authors suggested that absolute superiority of the approach will be apparent as more diverse non-European and admixed populations are included in genomic studies, as previously seen (Bitarello and Mathieson, 2020; Fatumo and Inouye, 2023; Márquez‑Luna et al., 2017). It is also worth noting that predictive accuracy of a PRS model for admixed individuals increases with an increasing proportion of European ancestry in that cohort and in that sense, the opposite is equally true (Bitarello and Mathieson, 2020; Cavazos and Witte, 2021). Using a European cohort as one of the training data sets, was not necessarily adequate as selection of a training cohort (e.g., for linear combination of PRS) was directly dependent on the ancestral contribution percentage in the target admixed population (Cavazos and Witte, 2021). In other words, increased predictive power could be achieved by using a cohort from the population that has the highest ancestry proportion in the target admixed population. These studies show that combining variants from European or African cohorts with a training cohort similar to the target population improves predictive accuracy (Fatumo and Inouye, 2023).

There are however inherent limitations of these findings, in that genetic data were from a two-way admixed population, had some level of European ancestry, and used simulated data that assumed not only similar genetic architecture but also shared SNPs effect size across cohorts. This could be a restrictive factor if the admixed target population understudy has either little to no proportion of European ancestry or exhibit a more complex admixture, as is the case with the South African multi-way admixed population group (Chimusa et al., 2014). Additionally, the partial PRS approach does not take into consideration the LD across ancestral segments and this has been known to skew overall PRS results and limit transferability. To circumvent this, a newer method and alternative for PRS construction, namely GAUDI, was developed (Sun et al., 2024). This method uses a fused lasso penalized regression framework specifically designed for admixed individuals to jointly estimate ancestry-specific effects. In the GAUDI framework, variants firstly undergo selection using clumping and thresholding (similar to the traditional PRS approach). Then, variant effects are penalized by balancing both fusion and sparsity components (as part of the fused lasso framework). The fusion component encourages similar ancestry-specific effects for the same variant, and the sparsity component ensures inclusion of variants with non-zero effect sizes. By incorporating both components, fused lasso regression promote a more structured and potentially generalizable model.

The superiority of GAUDI was demonstrated in predicting disease outcome, over other methods such as C + T, partial PRS and PRS-CSx (increased accuracy by > 60%) (Sun et al., 2024). The new method was more efficient as it uses a smaller number of variants to achieve similar or better results than other tools. This is advantageous; however, this feature also highlights the methods’ limitations. In fact, LD clumping is not optional while using GAUDI, but rather mandatory as the software tool cannot handle large numbers of variants to analyze simultaneously. Therefore, improvement of computational efficiency as well as algorithm development for parameter estimation present a crucial area for future research.

### Polygenic risk prediction of complex diseases today: application in real data

As of April 22, 2024, there were over 597 publications from which emanated 4723 published PRS (with performance assessed using (AUC)) on 652 traits and their subtypes (this information is regularly updated and can be found on the PGS Catalog website (https://www.pgscatalog.org/)). Most of those PRS studies have been conducted on predicting risks of neurology/neurodegeneration, cardiovascular diseases, and cancers, and have highlighted the potential of PRS for clinical validity .

PRS potential clinical use relates to intervention that either help diagnosis or include changes to screening initiation or frequency. This is a particularly promising approach for autoimmune diseases which appear to follow common-disease-common-variants architecture. For instance, in Type 1 Diabetes (T1D), Sharp et al. (2019) have designed and validated a PRS with an AUC of 0.92, which shows promise in guided selection of new-borns for auto antibody screening and the classification of T1D and T2D in adulthood (Sharp et al., 2019). The latter being important to avoid incorrect treatments as well as reduce medical costs and morbidity. Similar to T1D, Abraham and colleagues have previously demonstrated that PRS for celiac disease have the potential to replace HLA typing in conjunction with, or guide serology; thus, providing greater clinical diagnostics (Abraham et al., 2014). The results validated PRS across four different datasets and obtained AUCs of roughly 0.87–0.9, which represent a model that has a high predictive accuracy. More recently, in juvenile idiopathic arthritis (JIA) which is a common cause of disability in children, Canovas et al., (2020) trained PRS to predict disease risk (AUC = 0.67) in a cohort from the United Kingdom (UK) (Cánovas et al., 2020). The results were obtained using 10-fold cross-validation and tested in independent cohorts from Australia and the United States of America (USA), which achieved comparable AUCs of 0.67 and 0.65, respectively. They then extended their analysis to JIA subtypes and observed for the most common subtype, a substantial increase in prediction performance in the UK cohort (AUC = 0.82), Australian cohort (AUC = 0.84), and US-based cohort (AUC = 0.70). These findings are of particular interest as diagnosis of JIA is currently purely clinical with no molecular tests to support it, which results in longer wait time to secure a diagnosis (Cánovas et al., 2020). Therefore, JIA-PRS may provide timely stratification of JIA cases and therefore enabling early access to appropriate care. PRS have also been explored in breast cancer studies and a thorough review have been provided by (Willoughby et al., 2019). Noticeably, across studies, the AUC for most of them are in the 0.6–0.7 range. Although these values are modest and therefore unable to stratify between breast cancer cases and controls, lifetime risk estimates for breast cancer in individuals in the tails of the distribution can be assessed. For instance, Mavaddat et al. (2019) showed that the overall lifetime risk of developing breast cancer was 32.6% for individuals whose PRS values fell within the top percentile of the distribution (Mavaddat et al., 2019). These results could therefore provide more specific risk information for breast cancer screening decision making.

While these findings represent a significant advancement in exploring the potential of PRS to predict disease outcomes, there is a notable gap in the literature regarding the use of genetic prediction for communicable diseases. To date, only one study has documented the application of GWAS-derived PRS in predicting tuberculosis (TB) susceptibility (Hong et al., 2017). In their study, Hong and colleagues discovered 10 SNPs associated with PTB and combined their effect sizes with conventional risk factors such as age, sex, and body mass index (BMI) to calculate PRS. Remarkably, the predictive score achieved an in-sample validated AUC of 0.80, indicating its potential in identifying individuals at high risk of PTB who would benefit from preventive measures; albeit larger samples would be needed to validate the findings.

The aforementioned studies have all contributed valuable insights into the genetic basis of various diseases and the potential clinical validity of PRS (i.e., the ability of PRS models to predict disease risk). However, the complexity of the genetic architecture and the multidimensionality of genetic and environmental contributions to disease phenotypes significantly challenge the clinical implementations of genetic PRS.

Researchers are increasingly establishing that genotype-phenotype relationships may vary across different ancestral populations. This population-dependent accuracy of PRS suggests a significant loss in prediction accuracy when transferring PRS to a different population, posing challenges for generalization and subsequent clinical utility of PRS (i.e., the tangible effect of using PRS on patient health outcomes) (Bitarello and Mathieson, 2020; Grinde, Brown, et al., 2019; International Schizophrenia Consortium et al., 2009; Marnetto et al., 2020; Márquez‑Luna et al., 2017; Martin et al., 2017, 2019).

Despite the theoretical advantages of using PRS in clinical settings, practical implementation faces challenges. The clinical utility of these scores is often limited because they primarily account for genetic factors while neglecting environmental and lifestyle influences that also play significant roles in disease development. This limitation therefore raises questions about their effectiveness in real-world applications (Jung et al. 2024; Koch et al. 2023).

### Polygenic risk scores in prediction of treatment outcomes

In contrast to disease genetic studies, which focus on uncovering the genetic basis of a trait/disease, pharmacogenomics (PGx) studies investigate how genetic variation influences a person’s response to medication, encompassing factors such as drug metabolism, efficacy, and potential side effects, all aimed at refining drug therapy. Genetic variants may typically alter how the body processes drugs (pharmacokinetics (PK), which involves absorption, distribution, metabolism, elimination (ADME)) or affect the way drugs interact with their targets or biological pathways (pharmacodynamics (PD)), thereby altering sensitivity to the drug’s effects (Relling and Evans, 2015). Similar to many complex traits, most drug responses are influenced by the cumulative effect of multiple genes, highlighting the polygenic nature of the measured outcomes (Crouch and Bodmer, 2020; McInnes et al., 2021; Muhammad et al., 2021; Roden et al., 2019). Consequently, understanding the impact of genetic variants on treatment responses could pave the way for the development of targeted approaches that will enable clinicians to make more informed decisions by choosing drugs that are more effective while minimizing the risk of adverse drug reactions (ADRs).

Multiple studies have explored the use of PRS to predict treatment outcomes or ADRs, with a focus on treatment of psychiatric disorders, followed by circulatory and digestive conditions, as well as cardiovascular pharmacological endpoints as highlighted in a recent review by (Johnson et al., 2022) and (Cross et al., 2022). However, although significant findings were reported in the majority of cases, most of them only provided information relating to whether PRS were significantly associated with the outcomes of interest, by assessing the overall performance of the PRS models using proportion of variance explained (e.g., R^2^) (Johnson et al., 2022). The idea being that if PRS are able to account for a vast proportion of variance explained, then their clinical validity could be implied. However, the contribution of PRS in explaining phenotypic variance in pharmacological endpoints remains low, illustrating the fact that effort in PGx studies must continue.

Although the concept of PRS show great promise in PGx, their full impact has not been explored yet. This may not be surprising given the challenges of using PRS with PGx endpoints, particularly regarding safety and efficacy. This may be due to difficulty in accurately defining patient’s responses to medications, which requires a high level of accuracy.

In addition, well-defined endpoints are required for PGx studies in order to compare uniformly treated individuals – such as detailed patient data within specific time frames related to certain clinical scales – that may only be available through patient-level clinical databases. Another point of concern is polypharmacy which poses a significant challenge (in interpreting results) since it increases the likelihood of drug-drug interactions and susceptibility to toxicity especially when there is underlying condition like kidney failure. This could well compromise estimates of genetic effects and skew PRS results.

### Overview of PRS-PGx methods

A large variety of methods are available for PRS construction and analysis in PGx and a systematic literature review of the findings have been performed by (Angela Siemens et al. 2022), (Johnson et al. 2022) and (Zhai et al. 2023). The general trajectory of these findings suggest that compared with PRS modelling in disease GWAS, PRS analysis in PGx GWAS with drug response endpoints (efficacy or safety) is more challenging and faces additionally unique challenges. These include: (1) the lack of knowledge about whether to use PGx GWAS, disease GWAS or both GWAS/variants in the discovery cohort for PRS construction, (2) the significantly smaller sample sizes in PGx GWAS compared to large disease cohorts, as well as the more complex statistical modelling for handling both prognostic and predictive effects simultaneously, and (3) issues related to the transferability of PRS across population groups.

There is a trade-off between choosing PGx and disease GWAS (summary statistics) data in the discovery cohort used to build PRS. Choosing disease GWAS data, which typically has a large sample size, usually provides large power for prognostic effect prediction, but low power for predictive effect (i.e., genotype-by-treatment interaction) prediction. In contrast, choosing PGx GWAS data, which typically has a relatively small sample size, usually provides lower power for prognostic effect prediction, but likely larger power for predictive effect prediction since PGx variants used for PRS construction are directly drug response related. Additionally, there is the issue of “missing heritability” when using disease GWAS SNPs in PRS PGx as it has been shown that those SNPs recover about half of the full heritability of a drug response in PGx GWAS (Zhai et al., 2022). Since the choice of which SNPs to include is a priori unknown, Zhai and colleagues proposed a novel approach, PRS-PGx-Bayes – a *Bayesian* framework (see section “Bayesian regression approach”) – that leverages both PGx and disease GWAS in the discovery cohort for improving drug response prediction (Zhai et al., 2022). Using simulated data (*n* = 1000, 5000, 10,000), they demonstrate that PRS-PGx-Bayes performs consistently better than other PRS methods (such as C + T, Lassosum, and PRS-CS ( R^2^ = 0.27 vs. 0.20, 0.20, 0.23; respectively)); especially when the PGx-based GWAS sample size is sample (Zhai et al., 2023). However, the authors highlight that the approach may not increase PRS prediction accuracy when the sample size of PRS GWAS is large enough.

In theory, more complicated models can be constructed for further increasing the PRS prediction performance. However, not only would they be more computationally extensive, but they may also face additional barriers in clinical interpretation and implementation.

## Conclusion

This literature review highlights the divers methodologies for calculating PRS and their critical roles in genomic medicine. We traced the evolution from traditional to more advanced approached like Bayesian and Frequentist penalized regression, emphasizing on how these methods enhance the predictive power of PRS while addressing the complexities of polygenic trats. A significant focus was placed on improving prediction accuracy for underrepresented populations and admixed individuals, underscoring the importance of developing specialized software and tailored methodologies to ensure effective application across diverse genetic backgrounds. Despite the advancements, considerable work remains, including the need for validation across various population groups and translating PRS findings into actionable clinical insights. While combining PRS with established clinical risk factors and environmental variables can improve prediction accuracy, further research is needed to optimise these integrations. As methodologies continue to evolve, it is essential to establish clear ethical, legal, and social guidelines that protect patient’s well-being, addressing concerns related to privacy and informed consent. Future research should prioritise inclusive frameworks that consider these ethical implications while enhancing the utility of the scores. Ultimately, while significant progress have been made, ongoing efforts are necessary to refine methodologies and ensure that PRS can effectively contribute to personalized medicine for all individuals. By addressing existing limitations and fostering collaboration among researchers, clinicians, and communities, we can unlock the full potential of PRS to improve health outcomes and advance precision medicine.

## Data Availability

No datasets were generated or analysed during the current study.
